# Depression among Low-Income Female Muslim Uyghur and Kazakh Informal Caregivers of Disabled Elders in Far Western China: Influence on the Caregivers’ Burden and the Disabled Elders’ Quality of Life

**DOI:** 10.1371/journal.pone.0156382

**Published:** 2016-05-31

**Authors:** Meiyan Wang, Bin He, Yuhuan Wang, Fuchen Wu, Xuefeng Chen, Wenting Wang, Xue Yang

**Affiliations:** Shihezi University School of Medicine, Shihezi, Xinjiang, China; Chiba University Center for Forensic Mental Health, JAPAN

## Abstract

**Background:**

Paying attention to and improving the mental health of the informal caregivers of disabled elders has become a global public health priority. This study focused on low-income female Uyghur and Kazakh informal caregivers of disabled elders residing in China’s far west. It investigated the prevalence of and the major related factors of depressive emotion.

**Methods:**

A cross-sectional study was performed from September 2013 to January 2014 in Shawan Prefectures, Tuokexun Prefectures, Bole Prefecture and Urumchi city. Shawan Prefecture has the highest proportion of Kazakhs, whereas Tuokexun Prefectures, Bole Prefecture and Urumchi city have the highest proportion of Uyghurs in Muslim ethnic Uygur and Kazakh communities. Xinjiang Uyghur Autonomous Region is located in remote western China; this area is approximately 3,105 km (1,929 miles) away from Beijing. A total of 444 female Uyghur and Kazakh informal caregivers of disabled elders participated in this study. The self-rating depression scale, the Zarit burden interview, and the SF-36 questionnaire were used to evaluate the state of caregiver depression, caregiver burden, and quality of life (QOL), respectively. Statistical analyses were performed using multivariate logistic regression analyses, correlation with Spearman’s rho and independent-sample t-tests; a P-value of <0.05 was considered statistically significant.

**Results:**

Up to 38.5% (n = 217) of informal caregivers reported having depression, whereas 61.5% (n = 273) of them reported a lack of depression. Age of disabled elders more than 60 years old, total hours spent on caring daily≥8h, duration of caring≥5 years, negative self-evaluation of health condition, having caregiver burden, elders’ medium degree of disability and elders’ heavy degree of disability had a higher risk of caregiver depression. By contrast, daughter/daughter-in-law of disabled elders; unemployed carers, family’s per capita income >US$235.48(1500 yuan), high social support, and high QOL of disabled elders were each associated with a lower risk of depressive emotion. Moreover, informal caregivers with depression obtained high care burden scores; at the same time, disabled elders who were looked after by caregivers with depression obtained low QOL scores.

**Conclusions:**

Our findings suggest that the demographics characteristics of informal caregivers, and caregiver burden, and the disabled elders’ degree of disability and QOL had the most significant correlation with depressive emotion among women informal caregivers. The results had a enlighten that these variables should be considered while planning interventions to improve depression of informal caregivers.

## Introduction

Disability has become a primary subject of investigation in aging research. However, consensus on the definition or measure of disability is lacking. Thus, the term is defined in this paper as a general decline in several physiological systems and is characterized by disability and diminished functional ability; consequently, such decline induces vulnerability to adverse outcomes, including falls, hospitalization, institutionalization, and death [1]. In this paper, we define disabled older people as individuals who are unable to look after themselves because of chronic illness, physical injury, or old age, and thus must rely on others to take care of them [2]. This group of population demands long-term care. However, disabled older adults are cared for at home by family members because of national government advocacy and the influence of traditional culture [3–5]. Family members provide any informal unpaid type of physical and emotional care for an ill loved one at home, and such family members are referred to as “informal caregivers” [6]. Informal caregivers generally provide caregiving support, including help in tasks related to the activities of daily living (ADL) (e.g., help with dressing, bathing, eating, and using the toilet) or in instrumental activities of daily living (IADL) (e.g., help with preparing hot meals, shopping for groceries, taking medication, or managing money) [7–8]. The contribution of informal caregivers in elderly care has become extremely important. However, taking care of an older adult with long-term, chronic, and complex health problems is an extremely onerous task, which imposes role strain, caregiver burden (i.e., physical, psychological, social life, and financil burden), and responsibility on informal caregivers [9–10]. Several academics have reported that informal caregivers deal with caregiver pressure and burden for a long period, and this situation may eventually trigger psychiatric ailments, such as a depressive disorder, anxiety, stress, and low life satisfaction [11–12]. A depressive emotion appears as the most determining factor of the physical health of the caregivers compared with the general population in global terms. This fact appears in higher rates in persons caring for older adults with dementia or behavior disorders [13–14]. Thus, paying attention to the mental health of family caregivers and improving their psychological well-being has become a global public health priority.

The global population is aging rapidly, and this aging phenomenon is occurring in China where the number of older individuals aged 65 years and above has reached 221 million; the older population accounts for 16% of the total Chinese population [15]. Xinjiang Uyghur Autonomous Region is located in remote western China; it is approximately 3,105 km (1,929 miles) away from Beijing. Its largest inhabitant minority groups are Uyghurs and Kazakhs; the proportion of the elderly population in these groups has reached 11.18% and within 4.36% by 2013 and within a year [16]. With an aging society, the scale and number of Uyghur and Kazakh disabled elders have also increased annually [17]. Given the unique culture and society of Uyghur and Kazakh and the absence of a strong social welfare regime and of any formal care support, the family pension continues to occupy the dominant endowment model. In this model, a family member personally cares for disabled elders in their homes. As part of the unique customs and habits of the Uyghur and Kazakh society, the males go to the labor market to earn money, whereas the females stay at home to take care of their families (i.e., performing housework and caring for children and elders). Therefore, in this nation, the bulk of the caring for elders is provided by female informal caregivers, such as wives, daughters, daughters-in-law and granddaughters [18]. In terms of gender, the preponderance of depression is higher in women than in men [19]. Women provide a greater amount of care and ultimately experience a higher level of burden and depression compared with men [20]. Yee and Schulz reported higher levels of burden and depression among female caregivers [21]. Moreover, Xinjiang Uyghur Autonomous Region is an economically underdeveloped area; furthermore, the per capita income of Uyghur and Kazakh is lower than the national per capita income [22]. In 2001, the World Health Organization confirmed that in low-income countries, poverty both at the community (macro) and individual (micro) levels was associated with poor mental health functioning [23]. Although the Chinese government has issued relevant policies in recent years, such as the establishment of a new type of rural social endowment insurance [24], informal caregivers continue to receive limited financial or physical assistance from the state. Thus, the prevalence and the major related factors of depression of low-income female Urghur and Kazakh informal caregivers of disabled elders must be investigated.

Researchers have discussed the relationship between depression and caregiver burden. They suggested that caregivers with a higher caregiver burden exhibited more depression [25–26]. However, the major and interactive effects of depressive emotion on caregiver burden, particularly in low-income and resource-limited settings in Xinjiang Uyghur Autonomous Region, are unclear. Quality of life (QOL) is another major variable of examination in older adults and their informal caregivers. QOL is a construct that encompasses health and functioning, socioeconomic status, psychological, emotional, and spiritual aspects, general health perceptions, and family [27]. Some researchers have clarified that the stress of caregivers influenced their QOL outcomes [28–29]. However, fewer studies have examined the effects of the informal caregivers’ depressive emotion on the QOL of disabled elders. Additionally, the interaction between the informal caregivers’ depression and the disabled elders’ QOL is unclear.

In our investigation of the factors associated with depression, we analyzed the general demographic characteristics of the caregiver–elder dyads and included the informal caregivers’ care burden and the disabled elders’ QOL as independent variables in the multivariate logistic regression model. We subsequently tested the effects of a depressive emotion on the informal caregivers’ care burden and the disabled elders’ QOL. We hypothesized that (1) family caregivers with a depressive emotion have higher caregiver burden scores, and (2) disabled elders cared for by depressed informal caregivers have lower QOL scores.

In this exploratory study, we (1) examined the prevalence and the related factors of depression among the female informal caregivers of disabled elders; (2) analyzed the correlation of the depressive emotion between the informal caregivers’ care burden and disabled elders’ QOL; and (3) investigated the influence of informal caregivers’ depressive emotion on informal caregivers’ care burden and the QOL among disabled elders.

## Methods

### Study design and setting

This study was conducted in Xinjiang Uyghur Autonomous Region, where Kazakhs and Uyghurs are two large inhabitant minority groups. Following a pilot study, we used a cross-sectional study design with approval from the ethics committee to explore our objectives. We employed a stepped-wedge cluster sampling in Shawan Prefectures, Tuokexun Prefectures, Bole Prefecture and Urumchi City from September 2013 to January 2014. Shawan Prefecture has the highest proportion of Kazakhs, whereas Tuokexun Prefectures, Bole Prefecture and Urumchi City have the highest proportion of Uyghurs.

### Inclusion and exclusion criteria of study participants

The research participants were the family caregivers of community-dwelling disabled elders. We confirmed the informal caregivers based on our identification of the disabled elders. We used the following inclusion criteria in defining the disabled elders: (1) disabled elders of Uyghur and Kazakh minorities aged 60 years and above; (2) Katz ADL scores higher than 17 points, which fit the degree of disability of elders (i.e., “light,” “medium,”, and “heavy”) [30]; (3) residing in the research area; and (4) Mini Mental State Examination (MMSE) scores higher than or equal to 27 points; this score indicated the lack of cognitive impairment [31]. By contrast, we used the following exclusion criteria: (1) elders on a waiting list for a nursing home or living in the nursing home or convalescent hospital; and (2) an elder with a life expectancy of <1 year because of a terminal illness. Each included disabled older participant was then asked to nominate one family member or friend as his or her primary informal caregiver, who knew the older adult best and who was undertaking most of the caregiver burden for the required care of the disabled elders. Informal caregivers would be included if (1) they were 18 years or older; (2) they were the nominated family member of disabled elders aged 60 years and above; (3) they assisted in or supervised the ADL (i.e., personal care, mobility, communication, and emotional and/or practical and financial support) of the included elders; and (4) they provided unpaid family care. However, informal caregivers would be excluded if they were cognitively impaired and incapable of completing the measurements. Moreover, disabled elders and their informal caregiver would be excluded if either of them was unwilling to participate in this research.

**Fig 1:**
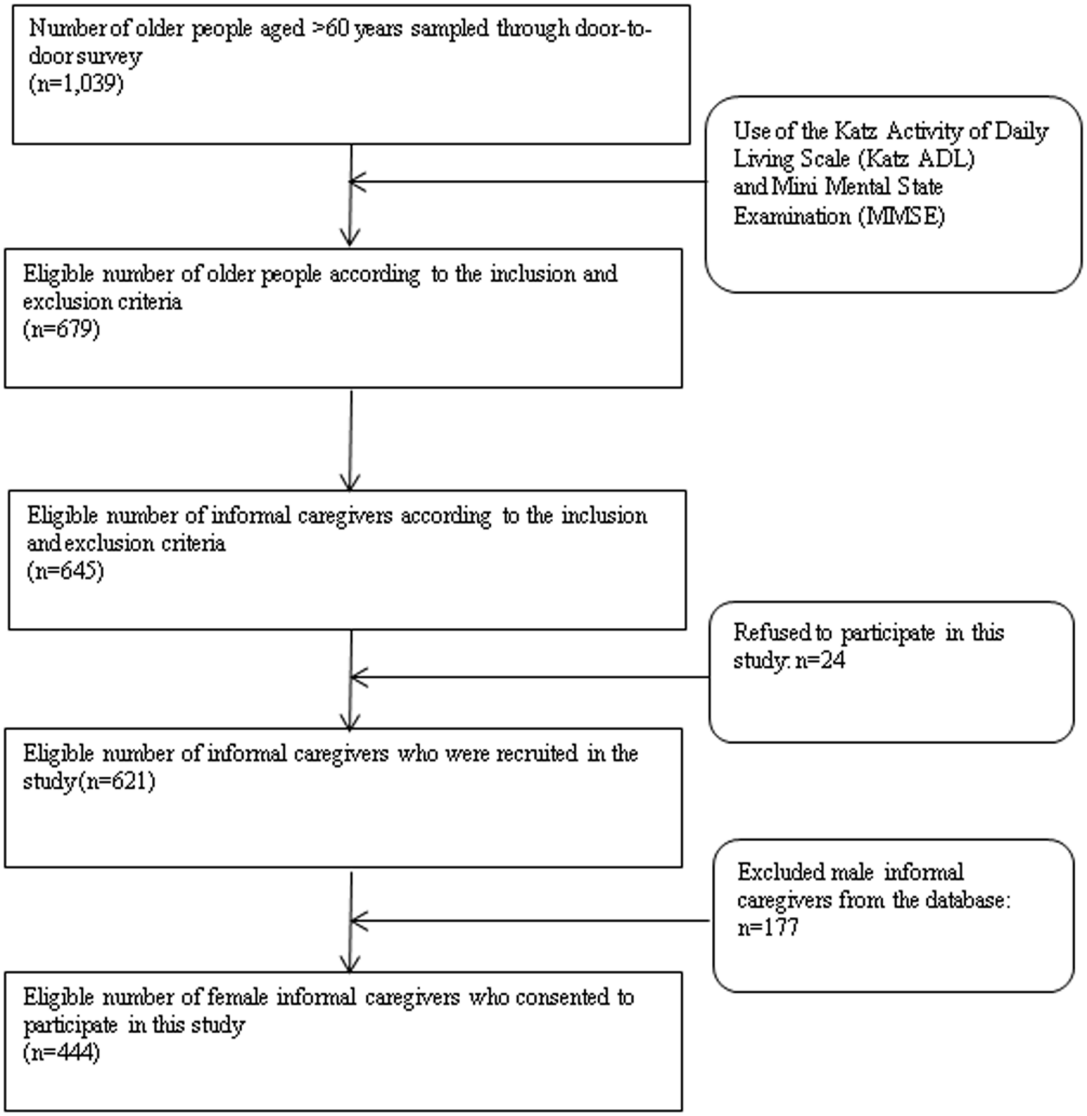
Recruitment of family caregiver participants.

The recruitment of family caregivers involved three steps. First, the disabled elders were selected and determined from the inclusion and exclusion criteria through the researchers’ door-to-door survey, under the assistance of community health institution staff members who profoundly know about the disabled elders’ basic situation. Second, these eligible elders nominated a primary informal caregiver who provided daily care. If the nominated informal caregivers were recruited from the inclusion criteria, then the family caregivers were invited to participate in this study. Finally, eligible informal caregivers who were interested in participating received an informed consent form, and their baseline measurements were recorded (see Fig 1).

### Assessment of caregivers’ depressive emotion

To evaluate participants’ depression, all of the informal caregivers filled out the Self-Rating Depression Scale (SDS) questionnaire. SDS was introduced by Doctor Zung in 1971. SDS is a 20-item inventory with a four-point Likert scale ranging from 1 (*never or a few times*) to 4 (*almost or all the time*), which evaluates the presence and severity of depressive symptoms. It measures four cognitive manifestations, namely, spirituality–emotional symptoms, somatic disorders, mental motility disorders, depression and mental disorder. An overall index ranging from 20 to 80 indicated the raw scores. The standard score was obtained through the raw scores multiplied by 1.25; higher scores indicated more severe symptoms. Additionally, the scores were grouped into several categories, namely, minimal(<50), mild (50–59), moderate (60–69), and severe symptoms (≥70) [32].

### Assessment of factors associated with caregiver depression

#### Demographic characteristics

Demographic characteristics were collected from informal caregivers through self-reports. The demographic characteristics included age, nation, educational level, marital status, relationship with the older resident, employment status, have children (yes vs. no), monthly family income (two categories: poor, ≤US$235.75 and good, >US$235.75), lives with the older resident (yes vs. no), total time spent on caring daily (hours), duration of caring (years), others helping to care for the elders (yes vs. no), self-evaluation of health condition (positive vs. negative), and social support (low vs. high). The demographic data of disabled elders were also assessed, including age, gender, nation, educational level, and degree of disability.

#### Social support of family caregiver

The social support of informal caregivers was measured by the Social Support Rating Scale (SSRS); SSRS was conducted by Xiaoshuiyuan [33], who relied on the situation of China (Cronbach’s alpha = 0.92). SSRS included 10 items and focused on three areas, namely, subjective support, objective support, and social support utilization. The total SSRS scores ranged from 0 to 66; a high score indicated a high level of social support. Scores less than 22 denoted a low level of social support, scores ranging from 22 to 44 indicated a moderate level of social support, and scores ranging from 45 to 66 suggested a high level of social support.

#### Family caregivers’ care burden

The reaction in the care burden of Uyghur and Kazakh informal caregivers as the primary outcome was measured using the Zarit burden interview (ZBI) [34,35]. ZBI consists of 22 items, with a five-point Likert scale ranging from 0 (*never*) to 4 (*nearly always*). It is widely used in appraising caregiver burden. The ZBI questions focus on the areas of caregivers’ health, psychological well-being, social life, and finances, as well as evaluate the overall burden [36]. The subscale used in this tool consists of four aspects, namely, personal strain, privacy conflict, guilt, and uncertain attitude. The total burden is obtained by summing the items to create a score from zero (lowest burden) to 88 (highest burden). No, mild, moderate, and severe burdens are scored at less than 20, 20–39, 40–59, and 60–88, respectively [37].

#### Degree of the elders’ disability and cognitive function

The degree of the elders’ disability was measured by Katz ADL at the baseline. The Katz ADL variable was categorized into three classes, namely, slight (unable to perform at least one of the six ADL tasks), moderate (unable to perform at least three of the six ADL tasks), and severe (unable to perform at least five of the six ADL tasks) [30]. To measure and describe the cognitive status of the disabled elders, MMSE as defined by Folstein was used [31]. The highest score was 30 points, scores in the normal range were 23 to 30, and scores <23denoted cognitive dysfunction.

#### QOL of the disabled elders

The SF-36 questionnaire was used to evaluate the QOL of disabled elders. It measures the eight concepts of physical functioning, bodily pain, role limitations due to physical, personal, and emotional health problems, emotional well-being, social functioning, energy/fatigue, and general health perceptions. Cronbach’s alpha is 0.824. The total score is obtained by summing the items to create a score from zero to 100; higher scores indicate a better QOL. Scores of more than 117, 72–117, and less than 72 denote good, moderate, and negative QOL, respectively [38, 39].

### Ethical statement

The Institutional Ethics Review Board (IERB) at the First Affiliated Hospital of Shihezi University School of Medicine granted ethical approval for the study (IERB No. SHZ201203801). Standard university hospital guidelines, including informed consent, voluntary participation, confidentiality, and anonymity, were followed. This study also complied with the culture of Muslim Uyghur and Kazakh. Prior to the study, all of the participants gave a written informed consent, including the information for the participants and their agreement on study participation. All of the personal data were treated as confidential and used only for scientific objectives.

### Statistical analysis

The statistical analysis involved third steps. In the first step, basic data variables that included the demographic characteristics, disabled elders’ QOL, and the depressive emotion and care burden of informal caregivers were summarized using a descriptive statistical presentation in percentages, means, and standard deviations. In the second step, the factors related to the depressive emotion of informal caregiver were examined through multivariate logistic regression analyses; the presence of depressive emotion was used as the dependent variable, whereas the demographic data of the informal caregiver and disabled elders, the disabled elders’ QOL, and the informal caregivers’ care burden were employed as independent variables. In the third step and final step were to suggest the association between the depression of informal caregivers and their care burden and disabled elders’ QOL: correlation with Spearman’s rho was performed to examine the relationship of the depressive emotion of informal caregivers with their care burden and disabled elders’ QOL; a t-test of independent means was employed to assess the effects of depression on the care burden of informal caregivers and the QOL of disabled elders. The statistical analysis was performed using the software program SPSS (version 17.0, 2008). A two-sided test probability level of less than 0.05 was applied and indicated statistical significance.

## Results

### Demographics characteristics of the study population

According to the selection criteria, 444 eligible healthy female Uyghur and Kazakh informal caregivers of disabled elders were retained from a database of 621 participants. The characteristics of the study population are summarized in Table 1. The ages of the eligible female informal caregivers ranged from 18 to 83 years, with a mean age of 41.32 years (SD = 14.49). The educational level of the carers is as follows: 67.1% (n = 298) completed secondary education, 24.3% (n = 108) completed primary education, and 8.6% (n = 38) had no education. Up to 92.6% of the carers (n = 411) were married, whereas 7.4% (n = 33) of the carers were single or separated. The relationship of the carers to the elders person is as follows: 32.4% (n = 144) of the carers were spouses, 65.3% (n = 290) of the carers were children (daughters or daughters-in-law), and 2.3% (n = 10) of the carers were other relatives. Roughly 64.2% (n = 285) of the carers had a family per capita income of less than 1,500 yuan (US$235.48). The majority of the informal caregivers worked part-time (62.4%, n = 277). A large number of informal caregivers (91%, n = 404) lived with the elders. Approximately 77.5% (n = 344) of informal caregivers had taken care of the elders for more than five years, whereas 22.5% (n = 100) of the carers had taken care of the elders for more than/less than five years. The majority of the carers (66.4%, n = 295) had others helping in the caring of the elders. Up to 66.7% (n = 296) of caregivers reported themselves to be in good health. The majority of the informal caregivers obtained a low level of social support. The average age of the disabled elders was 68.5 (SD = 7.83) years. Most of the disabled elders (75%, n = 333) lived in rural areas. The elders’ degrees of disability are as follows: 61% (n = 271) had a light degree of disability, 21.4% (n = 95) had a medium degree of disability, and 17.6% (n = 78) had a heavy degree of disability.

**Table 1:** Demographic characteristics of informal caregivers and disabled elders.

Characteristics	Family caregiver	Disabled elder
	N = 444	N = 444
Nation, n (%)		
Uyghur	244 (55)	244 (55)
Kazakh	200 (45)	200 (45)
Age (years), mean (SD)	41.32 (14.49)	68.5 (7.83)
Gender, n (%)		
Male		234 (52.7)
Female		210 (47.3)
Living area, n (%)		
Urban		111 (25)
Rural		333 (75)
Degree of disability, n (%)		
Light		271 (61)
Medium		95 (21.4)
Heavy		78 (17.6)
Educational level, n (%)		
None	38 (8.6)	139 (31.3)
Completed primary	108 (24.3)	180 (40.5)
Completed secondary	298 (67.1)	125 (28.2)
Marital status, n (%)		
Married or partnered	411 (92.6)	
Single or separated	33 (7.4)	
Relationship with the older resident, n (%)		
Spouse	144 (32.4)	
Daughter/daughter-in-law	290 (65.3)	
Other relatives	10 (2.3)	
Employment status, n (%)		
Full-time work	86 (19.4)	
Part-time work	277 (62.4)	
Unemployed	57 (12.8)	
Retired	24 (5.4)	
Have children, n (%)		
Yes	396 (89.2)	
No	48 (10.8)	
Per capita income (yuan), n (%)		
≤1,500	285 (64.2)	
>1,500	159 (35.8)	
Lives with the older resident, n (%)		
Yes	404 (91)	
No	40 (9)	
Total time spent on caring daily (hour), n (%)		
<8	88 (19.8)	
≥8	356 (80.2)	
Duration of caring (years), n (%)		
<5	100 (22.5)	
≥5	344 (77.5)	
Others’ help in caring for the elders, n (%)		
Yes	295 (66.4)	
No	149 (33.6)	
Self-evaluation of health condition, n (%)		
Positive	296 (66.7)	
Negative	148 (33.3)	
Social support, n (%)		
Low	427 (96.2)	
High	17 (3.8)	

### Prevalence of the depression and care burden of informal caregivers and the QOL of disabled elders

In this paper, according to our aim and the original cut-off score of questionnaire, we set the cut-off score of SDS was ≥50 (high score) as the threshold of having depression and cut-off score of SDS was ≤49 (low score)as the threshold of no depression; setting the cut-off score of ZBI was ≥20 (high score) as having burden and <20 (low score) as the no burden; setting the cut-off score of QOL was ≥72 (high score) as well QOL and the cut-off score of QOL was<72 (low score) as negative QOL.

The female informal caregivers’ mean score on the SDS was 48.45 (SE = 0.27). Up to 38.5% (n = 217) of caregivers reported having depression, whereas 61.5% (n = 273) of caregivers reported no depression. The mean ZBI total score of informal caregivers was 20.04 (SE = 0.43). Among the informal caregivers, 48.2% (n = 214) reported having caregiver burden, whereas 51.8% (n = 230) reported no caregiver burden. The disabled elders had a mean score on the SF-36 of 91.59 (SE = 0.58). The majority of the disabled elders (92.8%, n = 412) reported a high level of QOL, whereas 7.2% (n = 32) of the elders reported a low level of QOL (see Table 2).

**Table 2:** Prevalence and score on depression and care burden of informal caregivers and the quality of life of disabled elders.

Variable	Low level(%)	High level(%)	Scores Mean	SE
Family caregiver			48.45	0.27
Depression	61.5	38.5		
Care burden	51.8	48.2	20.04	0.43
Disabled elderly				
Quality of life	7.2	92.8	91.59	0.58

### Factors associated with caregiver depression

The results of the logistic regression analyses used to examine the effect of the factors on the presence of depressive emotion are presented in Table 3. Informal caregivers with age of 60 years and above had a 4.15 times higher risk of depression than those with age of less than 40 years. Caregivers with total hours spent on caring daily ≥8h and duration of caring ≥5 years had 3.35 times, 2.25 times higher risk of depression separately than those whose total hours spent on caring daily <8h and duration of caring <5 years, respectively. Depression of informal caregiver was significantly associated with elders’ degree of disability: compared with slight disability of elders, elders with medium and heavy disability had 3.23 times, 3.46 times higher risk of depression separately. Informal caregivers were also more likely to experience depression emotion if their self-evaluation of health condition were negative (2.30 times), had caregiver burden (2.76 times). While, caregivers with characteristics of daughter/daughter-in-law of the disabled elders had a 0.28 times lower risk of burden than those characteristics of spouse. As well as caregiver with unemployed (0.26 times), family per capita income >US$235.48(1,500 yuan) (0.24 times), high social support (0.05 times) and disabled elders’ high QOL (0.12 times) were each associated with a lowered risk of depressive emotion. A multivariate model that included these significant correlates could account for 33.2% of the variability among the depressive emotions experienced by informal caregivers (R^2^ = 0.332). No significant association was noted between the other characteristics of informal caregivers, such as educational level, marital status, having children, lives with the elders, and others’ help in caring for the elders. The characteristics of disabled elders, such as age, nation, gender, living area, and educational level, lacked a significant association with the informal caregivers’ depressive emotion.

**Table 3:** Multivariate logistic regression analysis: Factors associated with the depressive emotion among the informal caregivers of disabled elders (N = 444).

Variable	Odds ratio	95% CI	P-value
Informal caregivers’ characteristics (N = 444)			
<40	Ref.		
40–59	1.40	0.68–2.87	0.360
≥60	4.15	1.47–11.67	0.007
Educational level, n (%)			
None	Ref.		
Completed primary	1.25	0.45–3.49	0.669
Completed secondary	0.87	0.30–2.53	0.805
Marital status, n (%)			
Married or partnered	Ref.		
Single or separated	1.11	0.34–3.57	0.863
Relationship with the older resident, n (%)			
Spouse	Ref.		
Daughter/daughter-in-law	0.28	0.11–0.75	0.011
Other relatives	0.18	0.03–1.22	0.079
Employment status, n (%)			
Full-time work	Ref.		
Part-time work	0.58	0.23–1.41	0.228
Unemployed	0.26	0.08–0.82	0.022
Retired	0.53	0.15–1.91	0.333
Having children, n (%)			
Yes	Ref.		
No	1.58	0.58–4.36	0.373
Per capita income (yuan), n (%)			
≤1,500 (US$235.48)	Ref.		
>1,500 (US$235.48)	0.24	0.12–0.50	0.001
Lives with the older resident, n (%)			
Yes	Ref.		
No	1.30	0.52–3.26	0.575
Total time spent on caring daily (hour), n (%)			
<8	Ref.		
≥8	3.35	1.63–6.88	0.001
Duration of caring (years), n (%)			
<5	Ref.		
≥5	2.25	1.09–4.64	0.029
Others’ help in caring for the elders, n (%)			
Yes	Ref.		
No	1.18	0.69–2.00	0.545
Self-evaluation of health condition, n (%)			
Positive	Ref.		
Negative	2.30	1.39–3.82	0.001
Social support, n (%)			
Low	Ref.		
High	0.05	0.01–0.34	0.002
Caregiver burden, n (%)			
No	Ref.		
Having	2.76	1.63–4.68	0.001
Disabled elders’ characteristics (N = 444)			
Nation, n (%)			
Uyghur	Ref.		
Kazakh	1.13	0.64–2.00	0.671
Age (years)			
60–74	Ref.		
75–89	0.94	0.58–1.51	0.780
≥90	-	-	1.000
Gender, n (%)			
Male	Ref.		
Female	1.17	0.59–2.30	0.656
Living area, n (%)			
Urban	Ref.		
Rural	0.57	0.26–1.28	0.172
Degree of disability, n (%)			
Light	Ref.		
Medium	3.23	1.40–7.45	0.006
Heavy	3.46	1.35–8.85	0.010
Educational level, n (%)			
None	Ref.		
Completed primary	0.55	0.24–1.25	0.155
Completed secondary	1.11	0.57–2.18	0.757
Quality of life, n (%)			
Low	Ref.		
High	0.12	0.04–0.39	0.001

### Association between informal caregivers’ depressive emotion and care burden of informal caregivers and disabled elders’ QOL

The relationship between the informal caregiver’s depressive emotion (SDS total score) and the care burden of the caregiver (ZBI total score) and disabled elders’ QOL (SF-36 total score) was assessed (N = 444). Spearman’s correlation coefficients were conducted separately between the SDS total scores and the ZBI total scores and between the SDS total scores and QOL (SF-36 total score). The depressive emotion demonstrated a positive correlation with caregiver burden (*r =* 0.417) but a negative correlation with QOL (*r =* −0.175). The results are summarized in Table 4.

**Table 4:** Correlation with Spearman’s rho between the depressive emotion and the care burden of informal caregivers and the quality of life of disabled elders (N = 444).

Variable	Depression	
	Correlation coefficient	Significance (two-tailed)
Care burden of family caregiver	0.417	<0.001
Quality of life of disabled elders	−0.175	<0.001

A t-test of independent means was used in assessing the two hypotheses; the results are presented in Table 5. Caregivers with a depressive emotion reported significantly higher scores of care burden compared with caregivers without a depressive emotion. Caregivers with a depressive emotion scored 23.20 on care burden, whereas those without a depressive emotion scored 18.06, with a mean difference of −5.15. This result implied a significant difference in the care burden experienced by caregivers with a depressive emotion and those without a depressive emotion. Table 5 also shows that the disabled elders scored higher on QOL with their informal caregiver with no depressive emotion than those with their caregiver with a depressive emotion. The disabled elders among caregivers with no depressive emotion scored 92.94 on QOL, whereas those with a depressive emotion scored 89.44, with a mean difference of 3.50. This result confirmed a significant difference in the disabled elders’ QOL between their informal caregivers without a depressive emotion and with a depressive emotion.

**Table 5:** Tests of independent means in comparing effects of the lack and presence of depressive emotion on the care burden of informal caregivers and quality of life of disabled elders.

Variable	Depression	N	Mean	SD	Df	T-value	P-value
Family caregiver							
Care burden	No	273	18.06	8.225	442	−6.032	<0.001
	Having	171	23.20	9.525			
Disabled elders							
Quality of life	No	273	92.94	11.600	442	2.985	0.003
	Having	171	89.44	12.718			

## Discussion

Some studies have stated that the factors association with depression of female informal caregivers caring for disabled elders are differences significant in view of the diverse background of informal caregivers including nation, ethnicity and culture [40–41]. The Muslim population accounts for 24.3% of the global population[42], and this group have their own unique social culture and ethical principle[43]. However, fewer studies undertaken with the depression of Muslim Uyghur and Kazakh female informal caregivers have been evaluated previously. This paper demonstrated that 38.5% of female Uyghur and Kazakh informal caregivers of community-dwelling elders experienced depression. This rate is considerably higher than the recognized depression prevalence of 2.1% in the general adult population [44]. The current research noted a relative higher incidence of depression in the informal caregivers of disabled elders belonging to ethnic minorities. Thus, the mental health of the family caregivers of disabled elders should be given increased attention; at the same time, it’s necessary to develop an effective intervention to improve the family caregivers’ mental health.

The results of this study indicated that the depressive emotion of female informal caregivers of disabled elders was primarily associated with the caregivers’ demographics characteristics. The informal caregivers’ age and self-evaluation of health condition were each associated with an increased risk of depressive mood significantly. This result was concurred with that of Gurland et al. [45], who reported that the age of a caregiver was associated with both mental and physical health outcomes. This outcome could be attributed to the fact that most of the older caregivers of the disabled elders were spouses, and they along with the disabled elders suffered from various chronic diseases or functional disabilities, thereby increasing the caregivers’ stress. The results of this study confirmed that more total time spent on caring daily (hours) and longer duration of caring (years) worsen depression of caregivers. These results were consistent with those of Kuscul MK [46]. An endowment culture with national features exists within Xinjiang Uyghur and Kazakh minority groups during the long production process; this culture is based on the values of “honoring and respecting elders,” “helping and loving mutually,” and “being filial toward parents” [47]. Members of these minority groups have a duty to raise their children, and the children are responsible for caring for their aging parents at home. Family members would not send the disabled elders into nursing homes because of the influence of the endowment and religious cultures, in addition to being bounded by public opinions; thus, they insist on living with disabled elders [48]. They take care of the daily life and disease management for elders and help in the latter’s participation in the bulk of religious activities. Consequently, family caregivers looking after the elders invest more time and energy, thereby limiting their personal rest and social life time. At the same time, this situation triggers their own mental health problems in the long-term process of caring for disabled elders because of lack of relaxation and social support. An earlier research showed that higher levels of social participation could protect against caregiver burden and could be physiologically rewarding [49]. We also verified that caregiver burden that possibly caused depression from caregiver burden negatively influenced informal caregivers’ depression; this result concurred with previous reports [25–26]. Furthermore, the elders’ degree of disability was associated with a higher risk of the depression of informal caregivers. Informal caregivers would perform increased care work for disabled elders with moderate and serious degrees of disability. By contrast, we determined that being the daughter/daughter-in-law of the elders positively influenced the depression of informal caregivers. This result concurred with the finding that the informal caregivers’ age was the most negative factor in the caregivers’ depression. Filial piety is the most distinct characteristic in the traditional old pension culture. Parents have the supreme power in the family. Children should not defy their parents’ will and intentions. Children who do so are considered violators of family discipline; subsequently, they will be condemned and reviled by the people [50]. Hence, a situation in which the daughter/daughter-in-law cares for his/her disabled elders will not induce feelings of inhibition toward caring compared with a situation in which the spouse or other relatives are the caregivers. The high social support of caregivers and the high QOL of disabled elders were each associated with a lower risk of depression among informal caregivers significantly, which has implications for planning the intervention of improving the depression. Moreover, a higher family income positively influenced the caregivers’ depression. This result was consistent with that of Neri AL et al. [51], who reported that a low level of family income was a high-risk factor of depression. Therefore, our findings suggest that our government must be committed to developing the economy of ethnic minority areas to increase the household income for living expenses and increase the support for spending in medical equipment and welfare for elders and informal caregivers.

The results of this study confirmed the two hypotheses; that is, (1) a significant difference existed between the higher care burden scores among having depression of caregivers than those with no depression, and (2) disabled elders obtained lower QOL scores when cared for by caregivers with depression than when cared for by caregivers without depression. Therefore, we suggested that the association between the caregivers’ depression and their care burden and the disabled elders’ QOL could influence each other. Caregiver burden and disabled elders’ QOL have significant association with depression of informal caregivers, that are caregivers with a higher caregiver burden and low level of QOL among disabled elders increase depression of informal caregivers. On the contrary, it is possible that caregivers with depression are likely to feel more burdened and lower disabled elders’ QOL. Hence, we suspected that the depression of informal caregivers were possibly improved by alleviating their care burden and improving the disabled elders’ QOL. Conversely, improving the mental health of caregivers may reduce their care burden and improve the elders’ QOL.

Our research team had to overcome some difficulties in the process of collecting the data of caregivers and disabled elders. First, Xinjiang Uyghur Autonomous Region is extremely vast (i.e., covering 166 km^2^) and is characterized by an unbalanced economic development; thus, selecting a large sample with a good representative of Uyghur and Kazakh disabled elders became a key issue of this study. Therefore, we strictly adopted the stepped-wedge cluster sampling to ensure the representative object in this study based on our familiarity with the geographical distribution of Uyghur and Kazakh, and combined this aspect with the economic development and living standards of local residents. We subsequently determined the good sample selection. Second, the language of communication between the researchers and the participants became a problem in this study. Thus, we recruited the medical postgraduates with an understanding of bilingual double language (i.e., understanding the language of Han and Uygur, or understanding the language of Han and Kazak) and the local community health workers with bilingual double language. Third, research personnel training signified another key problem. Hence, after confirming the study protocol, all of the research personnel (including the medical postgraduates and local community health workers with understanding of bilingual double language) were required to undergo roughly four weeks of standardization training.

### Strengths and limitations of this study

The strength of our study is that our work is the first to focus on the depression of low-income Muslim Uyghur and Kazakh informal caregivers in western China; at the same time, it pioneers the examination of the prevalence and the related factors of depression among female informal caregivers of disabled elders. The results of this study supplement the literature on the depression of Uyghur and Kazakh informal caregivers of disabled elders. Another strength is the sampling used in this study.

The limitations of this paper include the use of a screening tool for depression rather than a structured diagnostic interview. This study also lacks sufficient samples to determine the independent effect of each informal caregiver problem. Furthermore, this study proved that the elders’ degree of disability could have a direct impact on caregiver depression. However, the sampling might have excluded the serious degree of disabled elders who were treated in the hospital because of their inclusion and exclusion criteria. This aspect might affect the detection prevalence and predictors of depression among informal caregivers.

## Conclusion

This study assessed the depressive emotion and care burden of female informal caregivers and the QOL of disabled elders. The prevalence rate of a depressive emotion was 38.5%, and the percentages of those with caregiver burden were 48.2% and 92.8% of disabled elders with high QOL. The informal caregivers’ demographic characteristics and the disabled elders’ degree of disability and QOL demonstrated the most significant correlation with the depressive emotion of female family caregivers of disabled elders. We also clarified that the caregivers’ high care burden and disabled elders’ low QOL could increase the caregivers’ depressive emotion, and high depressive mood could augment the caregivers’ care burden and disabled elders’ QOL. These variables should be considered in planning interventions to improve the depression of family caregivers.

## Supporting Information

S1 Fig Recruitment of family caregiver participants. (PDF)

S1 File First Affiliated Hospital of Shihezi University School of Medicine granted ethical approval. (PDF)

S1 Table Demographic characteristics of informal caregivers and disabled elders. (PDF)

S2 Table Prevalence and score on depression and care burden of informal caregivers and the quality of life of disabled elders. (PDF)

S3 Table Multivariate logistic regression analysis: Factors associated with the depressive emotion among the informal caregivers of disabled elders (N = 444). (PDF)

S4 Table Correlation with Spearman’s rho between the depressive emotion and the care burden of informal caregivers and the quality of life of disabled elders (N = 444). (PDF)

S5 Table Tests of independent means in comparing effects of the lack and presence of depressive emotion on the care burden of informal caregivers and quality of life of disabled elders. (PDF)

## References

[1] Aggar C, Ronaldson S, Cameron ID. Reactions to caregiving during an intervention targeting frailty in community living older people. BMC Geriatrics. 2012; 12: 66 10.1186/1471-2318-12-66 23095644 PMC3571864

[2] Gratão ACM, Talmelli LFDS, Figueiredo LC, Rosset I, Freitas CP, Rodrigues RAP. Functional dependency of older individuals and caregiver burden. Rev Esc Enferm USP. 2013; 47(1): 137–144. 23515813 10.1590/s0080-62342013000100017

[3] Navaie-Waliser M, Feldman PH, Gould DA, Levine C, Kuerbis AN, Donelan K. When the caregiver needs care: the plight of vulnerable caregivers. Am J Public Health. 2002; 92(3): 409–413. 11867321 10.2105/ajph.92.3.409 PMC1447090

[4] Carretero S, Garcés J, Ródenas F, Sanjosé V. The informal caregiver's burden of dependent people: theory and empirical review. Arch Geronto Geriatr. 2009; 49(1): 74–79. 10.1016/j.archger.2008.05.004 18597866

[5] Arno PS, Levine C, Memmott MM. The economic value of informal caregiving. Health Aff (Millwood). 1999; 18(2): 182–188. 10.1377/hlthaff.18.2.182 10091447

[6] Donelan K, Hill CA, Hoffman C, Scoles K, Feldman PH, Levine C, et al Challenge to care: informal caregivers in a changing health system. Health Aff. 2002; 21(4): 222–231. 10.1377/hlthaff.21.4.222 12117133

[7] Hammond T, Weinberg MK, Cummins R A. The dyadic interaction of relationships and disability type on informal carer subjective well-being. Quality of Life Research 2014; 23: 1535–1542. 24235087 10.1007/s11136-013-0577-4

[8] Hansen T, Slagsvold B, Ingebretsen R. The strains and gains of caregiving: an examination of the effects of providing personal care to a parent on a range of indicators of psychological well-being. Social indicators research. 2013; 114: 323–343.

[9] Mittelman MS, Ferris SH, Shulman E, Steinberg G, Ambinder A, Mackell JA, et al A comprehensive support program: effect on depression in spouse-caregivers of AD patients. Gerontologist. 1995; 35(6): 792–802. 8557206 10.1093/geront/35.6.792

[10] Mittelman MS, Roth DL, Coon DW, Haley WE. Sustained benefit of supportive intervention for depressive symptoms in caregivers of patients with Alzheimer’s disease. Am J Psychiatry. 2004; 161: 850–856. 15121650 10.1176/appi.ajp.161.5.850

[11] Mittelman MS, Haley WE, Clay OJ, Roth DL. Improving caregiver well-being delays nursing home placement of patients with Alzheimer disease. Neurology. 2006; 67: 1592–1599. 17101889 10.1212/01.wnl.0000242727.81172.91

[12] Ory MG, Hoffman RR, Yee JL, Tennstedt S, Schulz R. Prevalence and impact of caregiving: a detailed comparison between dementia and nondementia caregivers. Gerontologist. 1999; 39: 177–185. 10224714 10.1093/geront/39.2.177

[13] Robinson A, Lea E. Seeking respite: issues around the use of day respite care for the carers of people with dementia. Ageing and Society. 2011; 32: 196–218.

[14] Lopez-Hartmann M, Wens J, Verhoeven V, Remmen R. The effect of caregiver support interventions for informal caregivers of community-dwelling frail elderly: a systematic review. Int J Integr Care. 2012; 12(5): 133. 10.5334/ijic.845 PMC3601532 23593047

[15] The"twelfth five-year" plan for Chinese aging development. Available: http://www.chinanews.com.

[16] The national aging working committee office. Elderly population reached 2.53 million in xinjiang. 2014.6.18.

[17] Yang L, Qin JM. Health services survey in Xinjiangin. Xinjiang urumqi Health science and technology press. 2002.

[18] Wu FC, Wang YH, Chen XF. The relationship among the knowledge,attitude and practice of the main caregiver on caregiving the Uygur and Kazak’s disability elderly. Chinese Journal of gerontology. 2014; 35(6): 3100–3102.

[19] Kagotho N, Ssewamala FM. Correlates of Depression among Caregivers of Children Affected by HIV/AIDS in Uganda: Findings from the Suubi-Maka Family Study. AIDS Care. 2012; 24(10): 1226–1232. 10.1080/09540121.2012.658754 22375826 PMC3422693

[20] Pinquare M, Sorensen S. Gender differences in caregiver stressors,social resoures, and health:An updated meta-analysis.Journals of Gerontology:Series B. Psychological science and social science. 2006; 61(1): 33–45. 10.1093/geronb/61.1.p33 16399940

[21] Yee JL, Schulz R. Gender caregivers:A review and analysis. Gerontologist. 2000; 40(2): 147–164. 10820918 10.1093/geront/40.2.147

[22] Niu YF. The necessity and importance in accelerating the development of the economic and social for the minority nationality in xinjiang. The communist party of China (yili committee party school journal). 2010; 2: 41–43.

[23] WHO. The world health report 2001—Mental Health: New Understanding, New Hope. Geneva, Switzerland; 2001.

[24] Implementing new agricultural security coverage with 4.3 million farmers and herdsmen in xinjiang. Xin hua internet. 11Augst 2014. Available: http://news.xinhuanet.com/politics/2011-12/20/c_111258495.htm.

[25] Pinquare M, Sorensen S. Differences between caregivers and noncaregivers in psychological health and physical health. A meta-analysis. Psychology and Aging. 2003;18: 250–267. 12825775 10.1037/0882-7974.18.2.250

[26] Ferrell BR. End-of-life care:How well do we serve our patients?. Nursing. 1998; 28(9): 58–60. 9775888 10.1097/00152193-199809000-00024

[27] Ferrans C. Quality of life: Conceptual issues. Seminars in Oncology Nursing. 1990; 6: 248–254. 2274721 10.1016/0749-2081(90)90026-2

[28] Goode KT, Haley WE, Roth DL, Ford GR. Predicting longitudinal changes in caregiver physical and mental health: A stress process model. Health Psychology. 1998; 17: 190–198. 9548710 10.1037//0278-6133.17.2.190

[29] Schumacher KL, Dodd MJ, Paul SM. The stress process in family caregivers of persons receiving chemotherapy. Research in Nursing & Health. 1993; 16: 395–404. 8248566 10.1002/nur.4770160603

[30] Lawton MP, Brody EM. Assessment of older people:self-maintaining and instrumental activities of daily living. Gerontologist. 1969; 9: 179–186. 5349366

[31] Zung W W. A rating instrument for anxiety disorders. Psychossomatics. 1971; 12 (6): 371–379. 10.1016/S0033-3182(71)71479-0 5172928

[32] Folstein MF, Folstein SE, McHugh PR. A practical method for grading the cognitive state of patients for the clinician. J Psychiatr Res. 1975; 12(3): 189–198. 1202204 10.1016/0022-3956(75)90026-6

[33] Liu JW, Li FY, Lian YL. The reliability validation study of Social support rating scale. Journal of Xinjiang Medical University. 2008; 31(1): 1–3.

[34] Zarit SH, Reever KE, Bach-Peterson J. Relatives of the impaired elderly: Correlates of feelings of burden. Gerontologist. 1980;20: 649–655. 7203086 10.1093/geront/20.6.649

[35] Wang L, Yang XS, Hou Z, Feng QL, Wan-Jing YE. The application and evaluation on the Chinese version of caregiver burden scale. Chinese Journal of public health. 2006; 22: 970–972.

[36] Yang XD, Jiang YF. The commonly used assessment tool care burden for carers of patients with cerebral apoplexy. Chinese Journal of nursing. 2007; 42: 271–274.

[37] Toonsiri C, Sunsern R, Lawang W. Development of the burden interview for caregivers of patients with chronic illness. Journal of nursing and education. 2011; 4: 62–75.

[38] Ware JE, Sherbourne CD. The RAND-36 Short-form Health Status Survey: 1,Conceptual framework and item selection. Medical Care. 1992; 30(6): 473–481. 1593914

[39] Brazier E, Harper R, Jones NMB, O'Cathain A, Thomas KJ, Usherwood T, et al Validating the SF-36 health survey questionnaire: new outcome measure for primary care. BMJ. 1992; 305(6846): 160–164. 1285753 10.1136/bmj.305.6846.160 PMC1883187

[40] Holland JM, Thompson LW, Tzuang M, Gallagher-Thompson D. Psychosocial factors among Chinese American women dementia caregivers and their association with salivary cortisol: Results of an exploratory study. Ageing International. 2010; 35(2): 109–127.

[41] McCallum TJ, Spencer SM, Goins RT. Lost in summation: depression among african american female caregivers and noncaregivers. Journal of cross-cultural gerontology. 2008; 23(1): 77–84. 17912623 10.1007/s10823-007-9049-z

[42] Emerson M. Ethno-Religious Conflict in Europe:Typologies of Radicalization among Europe’s Muslim Communities Center for European Policy Studies. 2009.

[43] Goldberg A. “Islam in Germany”, in Hunter Shireen T.(ed.),Islam, Europe’s Second Religion:The New Sociol,Cultural,and Political Landscape, Westport, CT: Praeger, 2002; 29–50.

[44] Jenkins R, Lewis G, Bebbington P, Brugha T, Farrell M, Gill B, et al The national psychiatric morbidity surveys of Great Britain: initial findings from the household survey. Psychological Medicine. 1997; 27(04): 775–789. 9234456 10.1017/s0033291797005308

[45] Gurland B, Wilder DE, Berkman C. Depression and disability in the elderly: Reciprical relations and changes with age. International Journal of Geriatric Psychiatry. 1988; 3: 163–79.

[46] Kuscu MK, Dural U, Önen P, Yaşa Y, Yayla M, Basaran G, et al The association between individual attachment patterns,the perceived social support,and the psychological wellbeing of Turkish informal caregivers. Psychooncology. 2009; 18(9): 927–935. 10.1002/pon.1441 19140124

[47] Abulaiti AJ, Zhao FL. The study on the relationship between the traditional endowment of the Xinjiang minority nationality culture and the rural social old-age security in Xinjiang. Northwest Population Journal. 2009; 30: 84–88.

[48] Wimo A, Jönsson L, Bond J, Prince M, Winblad B, International AD. The worldwide economic impact of dementia 2010. Alzheimers & Dementia. 2013; 9(1): 1–11. 10.1016/j.jalz.2012.11.006 23305821

[49] Thompson HE Jr, Futterman A, Gallagher-Thompson D, Rose JM, Lovett SB. Social support and caregiving burden in family caregivers of frail elderly. Journal of Gerontology. 1993; 48: 245–254. 10.1093/geronj/48.5.s245 8366273

[50] Xinjiang foreign cultural exchange association making. Cole Croat folk culture. Xinjiang art photography press; 2006.

[51] Neri AL, Yassuda MS, Fortes-Burgos ACG, Mantovani EP, Arbex FS, de Souza Torres SV, et al Relations-hips between gender,age,family conditions,physical and mental health,and social isolation of elderly caregivers. International Psychogeriatrics. 2012; 24(3): 472–483. 10.1017/S1041610211001700 21929829

