# Supplementary figures and images for: Depression among Low-Income Female Muslim Uyghur and Kazakh Informal Caregivers of Disabled Elders in Far Western China: Influence on the Caregivers’ Burden and the Disabled Elders’ Quality of Life
Source: PLoS One. 2016 May 31;11(5):e0156382. doi: 10.1371/journal.pone.0156382 (PMC4887108; doi:10.1371/journal.pone.0156382)

**Figure 1. Recruitment of family caregiver participants.**

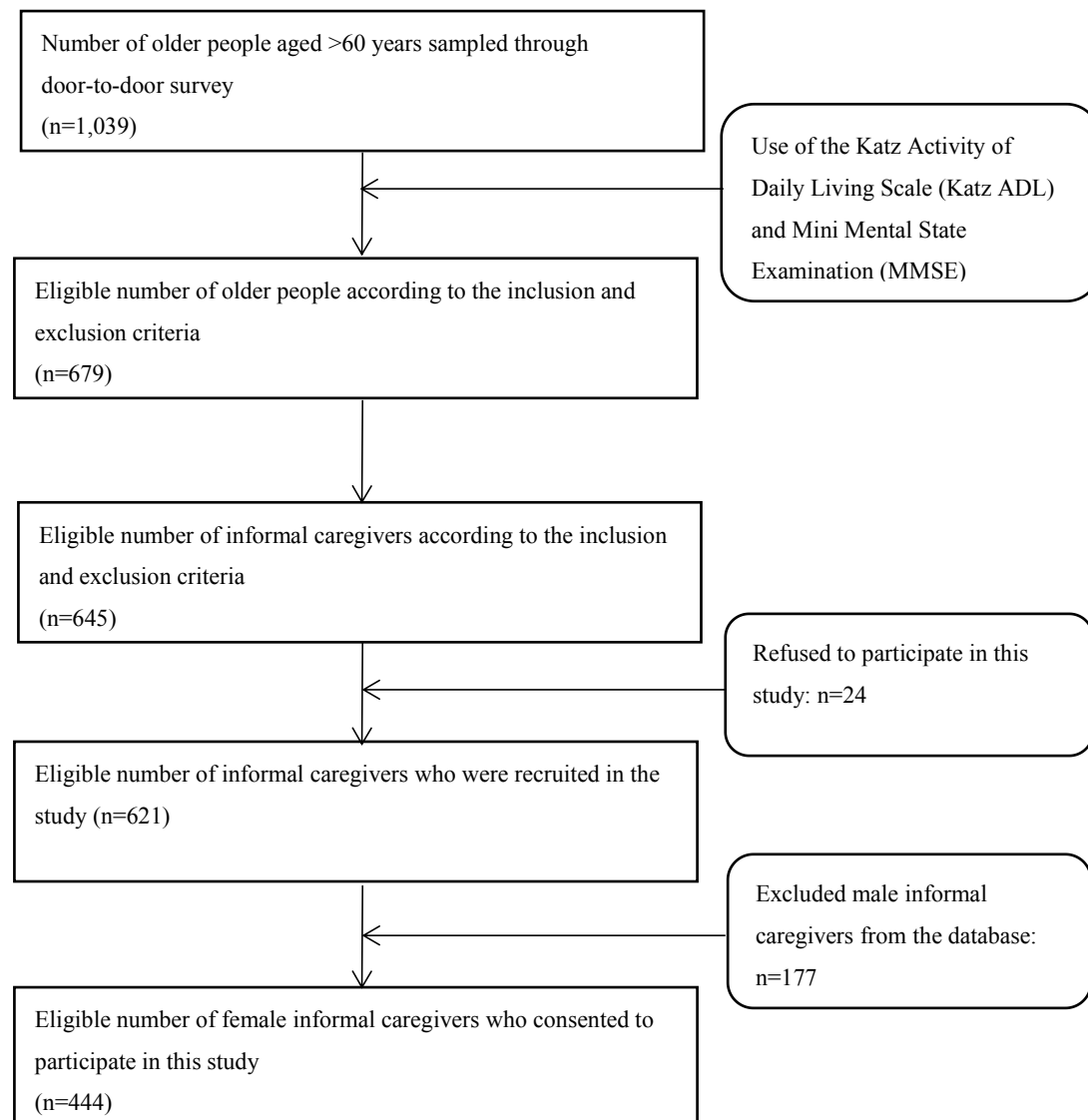

Supplement: S1 Fig — (PDF) [file pone.0156382.s001.pdf]
