# Supplementary material for: Depression among Low-Income Female Muslim Uyghur and Kazakh Informal Caregivers of Disabled Elders in Far Western China: Influence on the Caregivers’ Burden and the Disabled Elders’ Quality of Life
Source: PLoS One. 2016 May 31;11(5):e0156382. doi: 10.1371/journal.pone.0156382 (PMC4887108; doi:10.1371/journal.pone.0156382)
Supplement: S1 File — (PDF) [file pone.0156382.s002.pdf]

First Affiliated Hospital, Shihezi University School of Medicine

Institutional Review Board Approval

IRB ID：2012-038-01

Project Name	The study about Uygur, Kazak disability elderly family caregiver burden and intervention in Xinjiang		
Applicant Department	Shihezi University School of Medicine		
Principal Investigator	Yuhuan Wang	Title	Professor
Submitted Materials	Research Protocol	Yes■ No□	
	Observation Records	Yes■ No□	
	Researchers List	Yes■ No□	
Review Items	Researchers Qualifications	Qualified ■<br>Not Qualified □	
	Research Protocol	Appropriate ■<br>Not Appropriate □	
Valid Date	48months		

Review Approval：

The "The study about Uygur, Kazak disability elderly family caregiver burden and intervention in Xinjiang" study concerning the client's right to privacy. The study was approved by the Ethical Committee of the First Affiliated Hospital, Shihezi University School of Medicine, and was conducted according to the principles of the Declaration of Helsinki. Written informed consent was obtained from the subject, and his study considered Declaration of Helsinki as a statement of ethical principles.

Ethical Committee of the First Affiliated Hospital, Shihezi University School of Medicine

Date：2012-9-21
