# Supplementary material for: Depression among Low-Income Female Muslim Uyghur and Kazakh Informal Caregivers of Disabled Elders in Far Western China: Influence on the Caregivers’ Burden and the Disabled Elders’ Quality of Life
Source: PLoS One. 2016 May 31;11(5):e0156382. doi: 10.1371/journal.pone.0156382 (PMC4887108; doi:10.1371/journal.pone.0156382)
Supplement: S1 Table — (PDF) [file pone.0156382.s003.pdf]

Table 1. Demographic characteristics of informal caregivers and disabled elders.

Characteristics		Family caregiver	Disabled elder
		N=444	N=444
Nation, n (%)	Uyghur	244 (55)	244 (55)
	Kazakh	200 (45)	200 (45)
Age (years), mean (SD)		41.32 (14.49)	68.5 (7.83)
Gender, n (%)	Male		234 (52.7)
	Female		210 (47.3)
Living area, n (%)	Urban		111 (25)
	Rural		333 (75)
Degree of disability, n (%)	Light		271 (61)
	Medium		95 (21.4)
	Heavy		78 (17.6)
Educational level, n (%)	None	38 (8.6)	139 (31.3)
	Completed primary	108 (24.3)	180 (40.5)
	Completed secondary	298 (67.1)	125 (28.2)
Marital status, n (%)	Married or partnered	411 (92.6)	
	Single or separated	33 (7.4)	
Relationship with the older resident, n (%)	Spouse	144 (32.4)	
	Daughter/daughter-in-law	290 (65.3)	
	Other relatives	10 (2.3)	
Employment status, n (%)	Full-time work	86 (19.4)	
	Part-time work	277 (62.4)	
	Unemployed	57 (12.8)	
	Retired	24 (5.4)	
Have children, n (%)	Yes	396 (89.2)	
	No	48 (10.8)	
Per capita income (yuan), n (%)	≤1,500	285 (64.2)	
	>1,500	159 (35.8)	
Lives with the older resident, n (%)	Yes	404 (91)	
	No	40 (9)	
Total time spent on caring daily (hour), n (%)	<8	88 (19.8)	
	≥8	356 (80.2)	
Duration of caring (years), n (%)	<5	100 (22.5)	
	≥5	344 (77.5)	
Others' help in caring for the elders, n (%)	Yes	295 (66.4)	
	No	149 (33.6)	
Self-evaluation of health condition, n (%)	Positive	296 (66.7)	
	Negative	148 (33.3)	

Social support, n (%)	Low	427 (96.2)	
	High	17 (3.8)	
