# Supplementary material for: Depression among Low-Income Female Muslim Uyghur and Kazakh Informal Caregivers of Disabled Elders in Far Western China: Influence on the Caregivers’ Burden and the Disabled Elders’ Quality of Life
Source: PLoS One. 2016 May 31;11(5):e0156382. doi: 10.1371/journal.pone.0156382 (PMC4887108; doi:10.1371/journal.pone.0156382)
Supplement: S2 Table — (PDF) [file pone.0156382.s004.pdf]

Table 2. Prevalence and score on depression and care burden of informal caregivers and the quality of life of disabled elders.

Variable	Low level(%)	High level(%)	Scores	
			Mean	SE
Family caregiver			48.45	0.27
Depression	61.5	38.5		
Care burden	51.8	48.2	20.04	0.43
Disabled elderly				
Quality of life	7.2	92.8	91.59	0.58
