# Supplementary material for: Depression among Low-Income Female Muslim Uyghur and Kazakh Informal Caregivers of Disabled Elders in Far Western China: Influence on the Caregivers’ Burden and the Disabled Elders’ Quality of Life
Source: PLoS One. 2016 May 31;11(5):e0156382. doi: 10.1371/journal.pone.0156382 (PMC4887108; doi:10.1371/journal.pone.0156382)
Supplement: S3 Table — (PDF) [file pone.0156382.s005.pdf]

Table 3. Multivariate logistic regression analysis: Factors associated with the depressive emotion among the informal caregivers of disabled elders (N=444).

Variable		Odds ratio	95 % CI	P-value
Informal caregivers' characteristics (N=444)	<40	Ref.		
	40–59	1.40	0.68–2.87	0.360
	≥60	4.15	1.47–11.67	0.007
Educational level, n (%)	None	Ref.		
	Completed primary	1.25	0.45–3.49	0.669
	Completed secondary	0.87	0.30–2.53	0.805
Marital status, n (%)	Married or partnered	Ref.		
	Single or separated	1.11	0.34–3.57	0.863
Relationship with the older resident, n (%)	Spouse	Ref.		
	Daughter/daughter-in-law	0.28	0.11–0.75	0.011
	Other relatives	0.18	0.03–1.22	0.079
Employment status, n (%)	Full-time work	Ref.		
	Part-time work	0.58	0.23–1.41	0.228
	Unemployed	0.26	0.08–0.82	0.022
	Retired	0.53	0.15–1.91	0.333
Having children, n (%)	Yes	Ref.		
	No	1.58	0.58–4.36	0.373
Per capita income (yuan), n (%)	≤1,500 (US$235.48)	Ref.		
	>1,500 (US$235.48)	0.24	0.12–0.50	0.001
Lives with the older resident, n (%)	Yes	Ref.		
	No	1.30	0.52–3.26	0.575
Total time spent on caring daily (hour), n (%)	<8	Ref.		
	≥8	3.35	1.63–6.88	0.001
Duration of caring (years), n (%)	<5	Ref.		
	≥5	2.25	1.09–4.64	0.029
Others' help in caring for the elders, n (%)	Yes	Ref.		
	No	1.18	0.69–2.00	0.545
Self-evaluation of health condition, n (%)	Positive	Ref.		
	Negative	2.30	1.39–3.82	0.001
Social support, n (%)	Low	Ref.		
	High	0.05	0.01–0.34	0.002
Caregiver burden, n (%)	No	Ref.		
	Having	2.76	1.63–4.68	0.001
Disabled elders' characteristics (N=444)				
Nation, n (%)	Uyghur	Ref.		
	Kazakh	1.13	0.64–2.00	0.671
Age (years)	60–74	Ref.		
	75–89	0.94	0.58–1.51	0.780
	≥90	-	-	1.000
Gender, n (%)	Male	Ref.		

	Female	1.17	0.59–2.30	0.656
Living area, n (%)	Urban	Ref.		
	Rural	0.57	0.26–1.28	0.172
Degree of disability, n (%)	Light	Ref.		
	Medium	3.23	1.40–7.45	0.006
	Heavy	3.46	1.35–8.85	0.010
Educational level, n (%)	None	Ref.		
	Completed primary	0.55	0.24–1.25	0.155
	Completed secondary	1.11	0.57–2.18	0.757
Quality of life, n (%)	Low	Ref.		
	High	0.12	0.04–0.39	0.001
