# Supplementary material for: Depression among Low-Income Female Muslim Uyghur and Kazakh Informal Caregivers of Disabled Elders in Far Western China: Influence on the Caregivers’ Burden and the Disabled Elders’ Quality of Life
Source: PLoS One. 2016 May 31;11(5):e0156382. doi: 10.1371/journal.pone.0156382 (PMC4887108; doi:10.1371/journal.pone.0156382)
Supplement: S4 Table — (PDF) [file pone.0156382.s006.pdf]

Table 4. Correlation with Spearman's rho between the depressive emotion and the care burden of informal caregivers and the quality of life of disabled elders (N=444).

Variable	Depression	
	Correlation coefficient	Significance (two-tailed)
Care burden of family caregiver	0.417	<0.001
Quality of life of disabled elders	-0.175	<0.001
