# Supplementary material for: Depression among Low-Income Female Muslim Uyghur and Kazakh Informal Caregivers of Disabled Elders in Far Western China: Influence on the Caregivers’ Burden and the Disabled Elders’ Quality of Life
Source: PLoS One. 2016 May 31;11(5):e0156382. doi: 10.1371/journal.pone.0156382 (PMC4887108; doi:10.1371/journal.pone.0156382)
Supplement: S5 Table — (PDF) [file pone.0156382.s007.pdf]

Table 5. Tests of independent means in comparing effects of the lack and presence of depressive emotion on the care burden of informal caregivers and quality of life of disabled elders.

Variable	Depression	N	Mean	SD	Df	T-value	P-value
Family caregiver							
Care burden	No	273	18.06	8.225	442	-6.032	<0.001
	Having	171	23.20	9.525			
Disabled elders							
Quality of life	No	273	92.94	11.600	442	2.985	0.003
	Having	171	89.44	12.718			
